# 2-Amino-5-bromo­pyridinium hydrogen succinate

**DOI:** 10.1107/S1600536810006495

**Published:** 2010-02-27

**Authors:** Madhukar Hemamalini, Hoong-Kun Fun

**Affiliations:** a X-ray Crystallography Unit, School of Physics, Universiti Sains Malaysia, 11800 USM, Penang, Malaysia

## Abstract

In the title compound, C_5_H_6_BrN_2_^+^·C_4_H_5_O_4_^−^, the pyridine N atom of the 2-amino-5-bromo­pyridine mol­ecule is protonated. The protonated N atom and the amino group are linked *via* N—H⋯O hydrogen bonds to the carboxyl­ate O atoms of the singly deprotonated succinate anion. The hydrogen succinate anions are linked *via* O—H⋯O hydrogen bonds. A weak inter­molecular C—H⋯O hydrogen bond is also observed.

## Related literature

For background to the chemistry of substituted pyridines, see: Pozharski *et al.* (1997 ▶); Katritzky *et al.* (1996 ▶). For related structures, see: Goubitz *et al.* (2001 ▶); Vaday & Foxman (1999 ▶). For applications of succinic acid, see: Sauer *et al.* (2008 ▶). For bond-length data, see: Allen *et al.* (1987 ▶). For details of hydrogen bonding, see: Jeffrey & Saenger (1991 ▶); Jeffrey (1997 ▶); Scheiner (1997 ▶). For hydrogen-bond motifs, see: Bernstein *et al.* (1995 ▶).
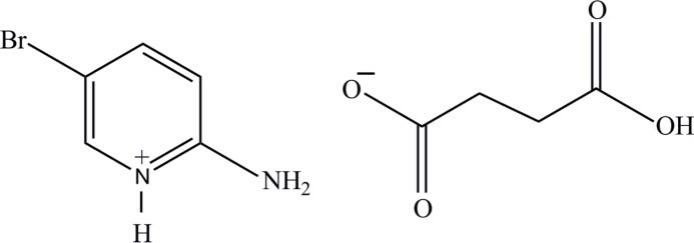


## Experimental

### 

#### Crystal data

C_5_H_6_N_2_Br^+^·C_4_H_5_O_4_^−^
*M* *_r_* = 291.11
Orthorhombic, 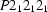

*a* = 5.3275 (2) Å
*b* = 13.6226 (5) Å
*c* = 15.1687 (5) Å
*V* = 1100.86 (7) Å^3^
*Z* = 4
Mo *K*α radiation
μ = 3.74 mm^−1^
*T* = 296 K
0.80 × 0.15 × 0.13 mm

#### Data collection

Bruker SMART APEXII CCD area-detector diffractometer
Absorption correction: multi-scan (*SADABS*; Bruker, 2009 ▶) *T*_min_ = 0.155, *T*_max_ = 0.650
10042 measured reflections
2472 independent reflections
2138 reflections with *I* > 2s(*I*)
*R*_int_ = 0.029

#### Refinement

*R*[*F* ^2^ > 2σ(*F* ^2^)] = 0.025
*wR*(*F* ^2^) = 0.057
*S* = 0.99
2472 reflections
150 parameters
H atoms treated by a mixture of independent and constrained refinement
Δρ_max_ = 0.21 e Å^−3^
Δρ_min_ = −0.31 e Å^−3^
Absolute structure: Flack (1983 ▶), 995 Friedel pairs
Flack parameter: 0.013 (8)

### 

Data collection: *APEX2* (Bruker, 2009 ▶); cell refinement: *SAINT* (Bruker, 2009 ▶); data reduction: *SAINT*; program(s) used to solve structure: *SHELXS97* (Sheldrick, 2008 ▶); program(s) used to refine structure: *SHELXL97* (Sheldrick, 2008 ▶); molecular graphics: *SHELXTL* (Sheldrick, 2008 ▶); software used to prepare material for publication: *SHELXTL* and *PLATON* (Spek, 2009 ▶).

## Supplementary Material

Crystal structure: contains datablocks global, I. DOI: 10.1107/S1600536810006495/is2526sup1.cif

Structure factors: contains datablocks I. DOI: 10.1107/S1600536810006495/is2526Isup2.hkl

Additional supplementary materials:  crystallographic information; 3D view; checkCIF report

## Figures and Tables

**Table 1:** Hydrogen-bond geometry (Å, °)

*D*—H⋯*A*	*D*—H	H⋯*A*	*D*⋯*A*	*D*—H⋯*A*
N1—H1*N*1⋯O2	0.85 (3)	1.88 (3)	2.720 (3)	171 (3)
N2—H2*A*⋯O1	0.86	1.92	2.782 (3)	178
N2—H2*B*⋯O1^i^	0.86	2.01	2.805 (3)	154
O4—H4⋯O2^ii^	0.82	1.85	2.609 (2)	154
C1—H1⋯O3^iii^	0.93	2.43	3.280 (3)	152

Symmetry codes: (i) 
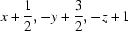
; (ii) 
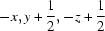
; (iii) 
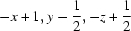
.

**Fig. 1.**
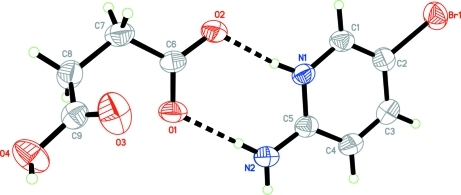
The asymmetric unit of the title compound. Displacement ellipsoids are drawn at the 50% probability level.

**Fig. 2.**
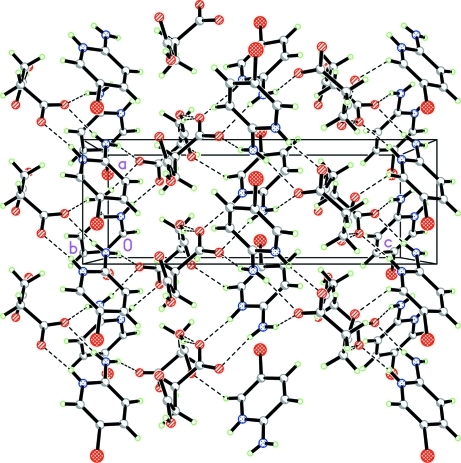
The crystal packing of the title compound, showing hydrogen-bonded (dashed lines) networks.

## supplementary crystallographic information

### Comment

Pyridine and its derivatives play an important role in heterocyclic chemistry (Pozharski *et al.*, 1997; Katritzky *et al.*, 1996). They are often involved in hydrogen-bonding interactions (Jeffrey & Saenger, 1991; Jeffrey, 1997; Scheiner, 1997).  Succinic acid derivatives are mostly used in chemicals, food and pharmaceuticals  (Sauer *et al.*, 2008). The crystal structures of 2-amino-5-bromopyridine (Goubitz *et al.*, 2001) and 2-amino-5-bromopyridinium propynoate (Vaday & Foxman, 1999) have been reported. In this paper, we present the X-ray single-crystal structure of 2-amino-5-bromopyridinium hydrogen succinate (I).

The asymmetric unit of (I) (Fig. 1) contains a 2-amino-5-bromopyridinium cation and a hydrogen succinate anion, indicating that proton transfer has occurred during the co-crystallization experiment. In the 2-amino-5-bromopyridinium cation, a wider than normal angle [122.9 (2)°] is subtended at the protonated N1 atom. The bond lengths (Allen *et al.*, 1987) and angles are normal.

In the crystal packing (Fig. 2), the protonated N1 atom and the 2-amino group (N2) is hydrogen-bonded to the carboxylate oxygen atoms (O1 and O2) *via* a pair of N—H···O hydrogen bonds, forming a *R*_2_^2^(8) ring motif (Bernstein *et al.*, 1995). The hydrogen succinate anions self-assemble *via* O4—H4···O2 (Table 1) hydrogen bonds.  Furthermore, the  crystal structure is stabilized by weak C—H···O hydrogen bonds, forming a 3D-network.

### Experimental

A hot methanol solution (10 ml) of 2-amino-5-bromopyridine (87 mg, Aldrich) and a hot aqueous solution  (10 ml) of succinic acid (59 mg, Merck) were mixed and warmed over a water bath for 10 minutes. The resulting solution was allowed to cool slowly at room temperature. Single crystals of the title compound appeared from the mother liquor after a few days.

### Refinement

Atom H1N1 was located in a difference Fourier map and refined freely. The remaining H atoms were positioned geometrically [C–H = 0.93 or 0.97 Å, O—H = 0.82 Å and N—H = 0.86 Å] and were refined using a riding model, with *U*_iso_(H) = 1.2 or 1.5*U*_eq_(C). 995 Friedel pairs were used to determine the absolute configuration.

### Crystal data

**Table:**

C_5_H_6_N_2_Br^+^·C_4_H_5_O_4_^−^	*F*(000) = 584
*M**_r_* = 291.11	*D*_x_ = 1.756 Mg m^−^^3^
Orthorhombic, *P*2_1_2_1_2_1_	Mo *K*α radiation, λ = 0.71073 Å
Hall symbol: P 2ac 2ab	Cell parameters from 4688 reflections
*a* = 5.3275 (2) Å	θ = 3.0–26.7°
*b* = 13.6226 (5) Å	µ = 3.74 mm^−^^1^
*c* = 15.1687 (5) Å	*T* = 296 K
*V* = 1100.86 (7) Å^3^	Needle, yellow
*Z* = 4	0.80 × 0.15 × 0.13 mm

### Data collection

**Table:**

Bruker SMART APEXII CCD area-detector diffractometer	2472 independent reflections
Radiation source: fine-focus sealed tube	2138 reflections with *I* > 2s(*I*)
graphite	*R*_int_ = 0.029
φ and ω scans	θ_max_ = 27.5°, θ_min_ = 2.0°
Absorption correction: multi-scan (*SADABS*; Bruker, 2009)	*h* = −6→6
*T*_min_ = 0.155, *T*_max_ = 0.650	*k* = −17→16
10042 measured reflections	*l* = −19→18

### Refinement

**Table:**

Refinement on *F*^2^	Secondary atom site location: difference Fourier map
Least-squares matrix: full	Hydrogen site location: inferred from neighbouring sites
*R*[*F*^2^ > 2σ(*F*^2^)] = 0.025	H atoms treated by a mixture of independent and constrained refinement
*wR*(*F*^2^) = 0.057	*w* = 1/[σ^2^(*F*_o_^2^) + (0.0248*P*)^2^] where *P* = (*F*_o_^2^ + 2*F*_c_^2^)/3
*S* = 0.99	(Δ/σ)_max_ = 0.002
2472 reflections	Δρ_max_ = 0.21 e Å^−^^3^
150 parameters	Δρ_min_ = −0.31 e Å^−^^3^
0 restraints	Absolute structure: Flack (1983), 995 Friedel pairs
Primary atom site location: structure-invariant direct methods	Flack parameter: 0.013 (8)

### Special details

**Table:**

Geometry. All s.u.'s (except the s.u. in the dihedral angle between two l.s. planes) are estimated using the full covariance matrix. The cell s.u.'s are taken into account individually in the estimation of s.u.'s in distances, angles and torsion angles; correlations between s.u.'s in cell parameters are only used when they are defined by crystal symmetry. An approximate (isotropic) treatment of cell s.u.'s is used for estimating s.u.'s involving l.s. planes.
Refinement. Refinement of F^2^ against ALL reflections. The weighted R-factor wR and goodness of fit S are based on F^2^, conventional R-factors R are based on F, with F set to zero for negative F^2^. The threshold expression of F^2^ > 2σ(F^2^) is used only for calculating R-factors(gt) etc. and is not relevant to the choice of reflections for refinement. R-factors based on F^2^ are statistically about twice as large as those based on F, and R- factors based on ALL data will be even larger.

### Fractional atomic coordinates and isotropic or equivalent isotropic displacement parameters (Å^2^)

**Table:**

	*x*	*y*	*z*	*U*_iso_*/*U*_eq_	
O1	0.0791 (3)	0.66915 (12)	0.38303 (10)	0.0490 (4)	
O2	0.2588 (3)	0.53970 (10)	0.32236 (11)	0.0441 (4)	
O3	0.0528 (4)	0.80559 (14)	0.20813 (14)	0.0633 (6)	
O4	−0.3210 (4)	0.86482 (12)	0.24424 (12)	0.0561 (5)	
H4	−0.2572	0.9169	0.2290	0.084*	
C6	0.0954 (4)	0.60694 (15)	0.32351 (14)	0.0341 (5)	
C7	−0.0907 (5)	0.61018 (17)	0.24855 (15)	0.0450 (6)	
H7A	−0.1850	0.5493	0.2485	0.054*	
H7B	0.0017	0.6134	0.1935	0.054*	
C8	−0.2747 (5)	0.69486 (17)	0.25115 (16)	0.0437 (6)	
H8A	−0.4068	0.6826	0.2085	0.052*	
H8B	−0.3515	0.6971	0.3091	0.052*	
C9	−0.1593 (5)	0.79274 (16)	0.23193 (13)	0.0377 (5)	
Br1	1.17196 (5)	0.336238 (18)	0.513704 (18)	0.05230 (10)	
N1	0.6002 (4)	0.52635 (13)	0.45518 (13)	0.0345 (4)	
N2	0.4388 (4)	0.66737 (14)	0.51595 (13)	0.0459 (5)	
H2A	0.3300	0.6690	0.4741	0.055*	
H2B	0.4388	0.7123	0.5558	0.055*	
C1	0.7639 (4)	0.45042 (15)	0.45213 (15)	0.0377 (5)	
H1	0.7547	0.4047	0.4067	0.045*	
C2	0.9404 (4)	0.44144 (16)	0.51537 (15)	0.0380 (5)	
C3	0.9540 (5)	0.51139 (18)	0.58345 (16)	0.0414 (6)	
H3	1.0756	0.5056	0.6271	0.050*	
C4	0.7899 (5)	0.58693 (16)	0.58521 (14)	0.0405 (5)	
H4A	0.7984	0.6332	0.6302	0.049*	
C5	0.6060 (4)	0.59588 (15)	0.51909 (14)	0.0343 (5)	
H1N1	0.482 (5)	0.5320 (17)	0.4180 (16)	0.047 (7)*	

### Atomic displacement parameters (Å^2^)

**Table:**

	*U*^11^	*U*^22^	*U*^33^	*U*^12^	*U*^13^	*U*^23^
O1	0.0616 (11)	0.0425 (9)	0.0428 (8)	0.0111 (9)	−0.0104 (8)	−0.0157 (8)
O2	0.0495 (10)	0.0309 (8)	0.0520 (9)	0.0067 (7)	−0.0090 (8)	−0.0090 (7)
O3	0.0470 (12)	0.0576 (12)	0.0852 (14)	−0.0024 (9)	0.0118 (11)	0.0229 (10)
O4	0.0603 (11)	0.0347 (9)	0.0733 (12)	0.0024 (10)	0.0199 (11)	0.0111 (8)
C6	0.0412 (13)	0.0265 (10)	0.0347 (11)	−0.0057 (9)	0.0013 (9)	−0.0001 (9)
C7	0.0572 (17)	0.0345 (12)	0.0432 (13)	−0.0017 (11)	−0.0075 (12)	−0.0049 (10)
C8	0.0426 (15)	0.0392 (12)	0.0493 (13)	−0.0033 (10)	−0.0088 (11)	0.0050 (10)
C9	0.0418 (13)	0.0385 (12)	0.0330 (10)	−0.0026 (12)	−0.0029 (12)	0.0039 (9)
Br1	0.04413 (14)	0.04024 (13)	0.07255 (17)	0.00556 (11)	−0.00191 (13)	0.00411 (12)
N1	0.0360 (11)	0.0308 (10)	0.0367 (10)	−0.0035 (8)	−0.0032 (9)	−0.0029 (8)
N2	0.0480 (11)	0.0377 (10)	0.0520 (10)	0.0049 (9)	−0.0099 (10)	−0.0151 (10)
C1	0.0415 (13)	0.0291 (11)	0.0426 (11)	−0.0051 (10)	0.0030 (10)	−0.0034 (9)
C2	0.0364 (12)	0.0334 (11)	0.0442 (11)	−0.0016 (9)	0.0024 (11)	0.0036 (10)
C3	0.0397 (13)	0.0436 (13)	0.0410 (12)	−0.0064 (12)	−0.0046 (11)	0.0035 (11)
C4	0.0443 (14)	0.0411 (12)	0.0362 (11)	−0.0048 (12)	−0.0015 (11)	−0.0057 (10)
C5	0.0355 (11)	0.0303 (10)	0.0370 (10)	−0.0069 (9)	0.0046 (10)	−0.0002 (9)

### Geometric parameters (Å, °)

**Table:**

O1—C6	1.241 (2)	N1—C1	1.354 (3)
O2—C6	1.264 (3)	N1—C5	1.356 (3)
O3—C9	1.199 (3)	N1—H1N1	0.85 (3)
O4—C9	1.319 (3)	N2—C5	1.321 (3)
O4—H4	0.8200	N2—H2A	0.8600
C6—C7	1.509 (3)	N2—H2B	0.8600
C7—C8	1.514 (3)	C1—C2	1.349 (3)
C7—H7A	0.9700	C1—H1	0.9300
C7—H7B	0.9700	C2—C3	1.407 (3)
C8—C9	1.497 (3)	C3—C4	1.351 (3)
C8—H8A	0.9700	C3—H3	0.9300
C8—H8B	0.9700	C4—C5	1.407 (3)
Br1—C2	1.891 (2)	C4—H4A	0.9300
			
C9—O4—H4	109.5	C1—N1—H1N1	121.6 (17)
O1—C6—O2	123.6 (2)	C5—N1—H1N1	115.4 (17)
O1—C6—C7	118.8 (2)	C5—N2—H2A	120.0
O2—C6—C7	117.60 (18)	C5—N2—H2B	120.0
C6—C7—C8	115.32 (18)	H2A—N2—H2B	120.0
C6—C7—H7A	108.4	C2—C1—N1	119.6 (2)
C8—C7—H7A	108.4	C2—C1—H1	120.2
C6—C7—H7B	108.4	N1—C1—H1	120.2
C8—C7—H7B	108.4	C1—C2—C3	119.8 (2)
H7A—C7—H7B	107.5	C1—C2—Br1	120.93 (17)
C9—C8—C7	114.1 (2)	C3—C2—Br1	119.31 (18)
C9—C8—H8A	108.7	C4—C3—C2	119.8 (2)
C7—C8—H8A	108.7	C4—C3—H3	120.1
C9—C8—H8B	108.7	C2—C3—H3	120.1
C7—C8—H8B	108.7	C3—C4—C5	120.2 (2)
H8A—C8—H8B	107.6	C3—C4—H4A	119.9
O3—C9—O4	123.3 (2)	C5—C4—H4A	119.9
O3—C9—C8	125.1 (2)	N2—C5—N1	118.3 (2)
O4—C9—C8	111.5 (2)	N2—C5—C4	123.99 (19)
C1—N1—C5	122.9 (2)	N1—C5—C4	117.7 (2)
			
O1—C6—C7—C8	3.3 (3)	C1—C2—C3—C4	0.2 (3)
O2—C6—C7—C8	−177.4 (2)	Br1—C2—C3—C4	179.88 (18)
C6—C7—C8—C9	70.8 (3)	C2—C3—C4—C5	−0.1 (3)
C7—C8—C9—O3	6.8 (3)	C1—N1—C5—N2	179.6 (2)
C7—C8—C9—O4	−173.26 (18)	C1—N1—C5—C4	−0.7 (3)
C5—N1—C1—C2	0.8 (3)	C3—C4—C5—N2	−180.0 (2)
N1—C1—C2—C3	−0.5 (3)	C3—C4—C5—N1	0.4 (3)
N1—C1—C2—Br1	179.82 (16)		

### Hydrogen-bond geometry (Å, °)

**Table:**

*D*—H···*A*	*D*—H	H···*A*	*D*···*A*	*D*—H···*A*
N1—H1N1···O2	0.85 (3)	1.88 (3)	2.720 (3)	171 (3)
N2—H2A···O1	0.86	1.92	2.782 (3)	178
N2—H2B···O1^i^	0.86	2.01	2.805 (3)	154
O4—H4···O2^ii^	0.82	1.85	2.609 (2)	154
C1—H1···O3^iii^	0.93	2.43	3.280 (3)	152

Symmetry codes: (i) *x*+1/2, −*y*+3/2, −*z*+1; (ii) −*x*, *y*+1/2, −*z*+1/2; (iii) −*x*+1, *y*−1/2, −*z*+1/2.

## References

[bb1] Allen, F. H., Kennard, O., Watson, D. G., Brammer, L., Orpen, A. G. & Taylor, R. (1987). *J. Chem. Soc. Perkin Trans. 2*, pp. S1–19.

[bb2] Bernstein, J., Davis, R. E., Shimoni, L. & Chang, N.-L. (1995). *Angew. Chem. Int. Ed. Engl.* **34**, 1555–1573.

[bb3] Bruker (2009). *APEX2*, *SAINT* and *SADABS* Bruker AXS Inc., Madison, Wisconsin, USA.

[bb4] Flack, H. D. (1983). *Acta Cryst.* A**39**, 876–881.

[bb5] Goubitz, K., Sonneveld, E. J. & Schenk, H. (2001). *Z. Kristallogr.* **216**, 176–181.

[bb6] Jeffrey, G. A. (1997). *An Introduction to Hydrogen Bonding.* New York: Oxford University Press.

[bb7] Jeffrey, G. A. & Saenger, W. (1991). *Hydrogen Bonding in Biological Structures.* Berlin: Springer.

[bb8] Katritzky, A. R., Rees, C. W. & Scriven, E. F. V. (1996). *Comprehensive Heterocyclic Chemistry II* Oxford: Pergamon Press.

[bb9] Pozharski, A. F., Soldatenkov, A. T. & Katritzky, A. R. (1997). *Heterocycles in Life and Society* New York: Wiley.

[bb10] Sauer, M., Porro, D., Mattanovich, D. & Branduaradi, P. (2008). *Trends Biotechnol.* **26**, 100–108. 10.1016/j.tibtech.2007.11.006 18191255

[bb11] Scheiner, S. (1997). *Hydrogen Bonding. A Theoretical Perspective* New York: Oxford University Press.

[bb12] Sheldrick, G. M. (2008). *Acta Cryst.* A**64**, 112–122. 10.1107/S0108767307043930 18156677

[bb13] Spek, A. L. (2009). *Acta Cryst.* D**65**, 148–155. 10.1107/S090744490804362X PMC2631630 19171970

[bb14] Vaday, S. & Foxman, M. B. (1999). *Cryst. Eng.* **2**, 145–151.

